# Views and experiences of eating disorders treatments in East Asia: a meta-synthesis

**DOI:** 10.1186/s40337-024-01070-4

**Published:** 2024-08-20

**Authors:** See Heng Yim, Ulrike Schmidt

**Affiliations:** 1 South London and Maudsley NHS Foundation Trust, London, UK; 2 Institute of Psychiatry, Psychology and Neuroscience, Department of Psychological Medicine, King’s College London, De Crespigny Park, Box P059, London, SE5 8AF UK

**Keywords:** Eating disorder, Meta-synthesis, East Asia, Psychological therapy, Anorexia nervosa

## Abstract

**Introduction:**

Although there have been qualitative meta-syntheses on experiences of eating disorders treatments, there is a paucity of syntheses specifically examining the perspectives and experiences of eating disorders treatments (ED) in East Asia (EA). Such synthesis could facilitate a better understanding of culture-specific perspectives and experiences. This review complements a quantitative scoping review published on ED treatments in EA (Yim & Schmidt, 2023), where most interventions reviewed focused on cognitive behavioural therapy (CBT) and internet interventions. The present meta-synthesis summarises stakeholders’ views on treatments and to synthesise clinical and research recommendations.

**Method:**

A systematic search of five databases and a citation search were conducted to identify relevant studies and data were analysed using thematic synthesis. Out of the 301 studies found, a total of 12 papers were included in the analysis.

**Results:**

A diverse range of treatments, such as family therapy, paediatric/psychiatric inpatient care, CBT, and counselling, were discussed. Three overarching themes were identified: Delineating Physical and Psychological Recovery; ‘I am not alone in this battle’; and Barriers to Change. The themes further delve into the various obstacles to recovery, including financial concerns and limited access to professionals and services. Culture-specific factors include family obligations and promoting family harmony. Balancing interdependence and independence from one’s family, as well as understanding family body ideals versus broader societal body ideals, are important considerations in ED interventions.

**Discussion:**

Some themes paralleled other qualitative syntheses, highlighting improved family relationships, perceived authoritarianism in treatments, and financial barriers. The review extends beyond the previous findings, revealing nuanced factors like family roles, cultural values, and norms. Clinical recommendations include incorporating family context in treatment and considering cultural influences on body image ideals. Capacity building through telemedicine and increased training is essential for advancing ED treatment in East Asia. Continued research is needed to better understand and treat people affected by ED in EA.

**Supplementary Information:**

The online version contains supplementary material available at 10.1186/s40337-024-01070-4.

## Abstract (keypoints)

Research on eating disorders (EDs) treatment mainly focuses on Western countries, with little exploration of experiences in East Asia. To fill this gap, we reviewed 12 studies on the perspectives of individuals, families and clinicians regarding EDs treatments in East Asia. Our synthesis identified three main themes:

*Physical and Psychological Recovery*: Effective treatment needs to address both physical and mental aspects of recovery.

*Finding Support – ‘I am not alone in this battle’*: Many individuals find strength in knowing they are not alone.

*Barriers to Change*: Obstacles like financial difficulties, limited EDs knowledge from professionals, and cultural factors can hinder recovery.

Cultural-specific factors such as family obligations and maintaining family harmony can impact on treatment motivation and effectiveness. Balancing family’s wishes/ interdependence and personal aspirations/ independence can also be a challenge. Our findings highlight the need for culturally sensitive treatments. Expanding telemedicine and increasing provider training can also help overcome treatment barriers. In conclusion, understanding cultural and contextual factors is essential for developing effective support systems and improving ED treatment outcomes in East Asia.

**Supplementary Information:**

The online version contains supplementary material available at 10.1186/s40337-024-01070-4.

## Introduction

Eating disorders (EDs) research has been historically centred in the Global North. However, in the past decade, more attention has been paid to non-WEIRD (Western, Educated, Industrial, Rich, Democracies) populations. Reviews report an increasing incidence of EDs in regions in East Asia (EA), where the prevalence of EDs may now be comparable to that reported in North America or Europe [1]. Young females are seen as an at-risk population for developing EDs in almost every country in EA. The exceptions include North Korea, because as of 2023, there have been no studies conducted in that country. There is also no epidemiological study on EDs in Mongolia [2]. Chen et al. [2] also report that in China, the prevalence of binge eating disorder (BED) exceeds that of bulimia nervosa (BN), with both being higher than the prevalence of anorexia nervosa (AN).

Different cultural factors have been hypothesised to contribute to the development and maintenance of EDs. Previous studies suggest that self-construal, which refers to how individuals define themselves in terms of independence from or interdependence with others, could influence treatment seeking when experiencing psychological distress [3]. Asians, who often endorse collectivistic values and exhibit interdependent self-construal, may articulate treatment goals in terms of benefiting their family. Another relevant concept is family harmony, particularly emphasised in ethnic Chinese culture, the largest ethnic group in EA. Values such as promoting interpersonal and family harmony and ‘saving face’ are seen as important and may affect help-seeking behaviours [4]. Contemporary EA societies face conflicting collectivistic and individualistic values where people grapple with bicultural contextual forces. Negotiating these conflicting value systems can lead to identity conflict, potentially serving as risk factors for body dissatisfaction and disordered eating [5]. Dysfunctional psychological individuation, the process of developing a sense of self and transitioning from hierarchical to symmetrical (more equal relationship between equal adults) parent-child relationships, is linked to the development of mental health conditions [6]. Additionally, values such as filial piety may hinder the process of individuation from the family [7].

Body image disturbance has been a core diagnostic criterion in the West. Research on body image dissatisfaction in EA has been mixed. Sing Lee [8] identified the presence of non-fat-phobic AN in EA, differentiating from the EDs phenotype in the West. Other studies have consistently identified high drive of thinness and body dissatisfaction in countries such as China [9] and South Korea [10]. Whilst earlier studies suggested that Westernisation is a factor in body image disturbance in EA [11], other researchers have challenged these findings. A 12-month prospective study found that Asian women reported more pressure and body comparison from social media depictions from Asian media when compared to Western media, suggesting that Asian media influences were more salient [12]. Other cross-cultural studies identified that Chinese American students have less body dissatisfaction than other American students [13]. That said, the impact of Westernisation may be reflected in the racialisation of body, where Asian Americans may be more distressed by certain body parts such as the shape of their eyes/nose, or their breast size [14]. Although it is difficult to directly compare East Asians living in the diaspora or as international students with those East Asians that are residing in their home countries, the research findings point to the nuanced influence of Westernisation on body ideals.

Policy, alongside cultural norms, can significantly influence individuals’ mental health and recovery. China’s historical One Child Policy (OCP) has been a focal point of research, examining how the policy led to an imbalanced gender ratio in China with more males than females, as well as how being an only child may impact social development compared to having siblings. Some studies suggest that only children may exhibit more self-centred and competitive behaviours [15], others report contradictory findings. For instance, Settles et al. [16] referenced the heightened pressure from parents onto their only children to excel academically, equating academic success with overall success [16]. Additionally, the systemic devaluation of females is evident, as seen in Zhejiang Province, China, where couples were allowed a second child only if the first child was a girl.

Yim & Schmidt [17] conducted a systematic scoping review on psychological treatments for EDs in EA. Compared to Europe and North America, there were significantly fewer EDs intervention studies. Out of the 18 published studies, most were feasibility or uncontrolled studies, but they generally showed good intervention acceptability and positive effects on ED symptoms. Notably, cognitive therapies were the predominant approach used, with family therapy largely absent in the literature despite being a first-line treatment for EDs in countries like the UK [18]. Qualitative studies in EA can complement quantitative findings. For instance, [7] described a culturally-adapted family therapy model based on the Micucci [19] approach. This model views the family’s response to the illness as a symptomatic cycle and aims to address family conflicts, including marital issues, which distinguishes it from ED-focused family therapies like Family-based treatment (FBT) and the Maudsley model (FT-ED). Additionally, the model focuses on promoting individuation of the young person from their family. Tan et al. [20] described the most helpful family involvement in the Asian context would be maternalistic, where family is a supportive, caring and loving, rather than paternalistic, which is seen as taking control of the decisions. Yim & Schmidt [17] also reported structural adaptations of EDs treatments such as having shortened treatment sessions for practical reasons, where healthcare is not free and specialist centres are far away in some regions in EA.

Qualitative synthesis provides a richer understanding that goes beyond understanding the effects of interventions on symptoms, and include stakeholders’ views, perceptions and experiences of treatments. To our knowledge, there is no qualitative synthesis of EDs treatment experiences nor professionals’ views of ED treatments in EA. A previous synthesis looked at experiences of family-based treatment (FBT) for AN among adolescents [21]. Themes such as relinquishing control ambivalently (initial treatment resistance, authoritative care), improved family relationships, and failure to address family issues were identified. Such synthesis can facilitate a better understanding of culture-specific perspectives of all stakeholders, which may lay a foundation for hypothesis-generation and testing in future EDs interventions research. Hence, the aim of this review is to synthesise the views and experiences of patients, families and healthcare professionals of EDs treatments in EA, with a particular focus on the cultural aspects influencing treatments.

## Method

The search was conducted according to the Enhancing Transparency in Reporting the synthesis of Qualitative research (ENTREQ) statement [22]. The search strategy was devised in consultation with a specialist librarian, and included both a database and citation search. Four English databases were comprehensively searched: Embase, Global Health, Ovid Medline, APA PsycINFO (any time till June 2024). As researcher SHY also understands Chinese, the Chinese research database was also searched (https://oversea.cnki.net/kns/defaultresult/index) with the search term eating disorders (饮食/进食失调) using subject headings search. Search terms were (eating disorder* or bulimia or anorexia or binge eating or disordered eating or ARFID or Avoidant Restrictive Food Intake Disorder) AND (China or Hong Kong or Taiwan or Macau or Macao or Mongolia or Japan or Korea or Chinese or Taiwanese or Mongolian or Japanese or Korean or east Asia or east Asian or far east) AND (qualitative or interview). Keyword search and subject heading search together with title/abstract search was done (see supplementary info for an example of search string).

*Inclusion criteria*.

- Peer-reviewed qualitative studies on the views, experiences or perceptions of EDs interventions, from service providers, patients, or families in East Asia. Regions in East Asia include China, Hong Kong, Japan, Macau, Mongolia, North Korea, South Korea and Taiwan (Asia Society, https://asiasociety.org/countries-regions/east-asia).
- Articles published in English or Chinese.

*Exclusion criteria*.

- Studies on the East Asian diaspora.
- Descriptive studies or single case study without a clear qualitative data collection and analysis methodology, clinical opinion papers.
- Books, dissertations, conference abstracts.

### Data analysis and extraction

Screening and deduplication were done on Rayyan software [23]. Thomas and Harden [24] thematic synthesis method was chosen for its suitability in understanding people’s views and experiences of EDs treatments to inform clinical practice, as opposed to developing theories or models like grounded theory. As no previous reviews existed in this area, integrating existing studies in a review was crucial for informing future clinical practice and research. Unlike quantitative meta-analysis, which focuses on prediction, this method emphasises interpretive explanations. In this study approach, although the data search was systematic, the purpose of study inclusion was purposive rather than exhaustive, aiming for conceptual understanding rather than data saturation. SHY independently conducted the screening of the texts and discussed any uncertainties with US.

The analysis proceeded in several steps. Firstly, the first author, SHY, familiarised herself with the papers. Themes and all participants’ quotes from both the *Results and Discussion* sections of each paper were then extracted and coded line-by-line using QSR NVivo [25]. Additional information such as participant demographics, diagnosis, and research method were also extracted to preserve study context. Codes were then grouped and categorised inductively based on their meanings, with attention paid to draw out culture-specific themes. The free codes were grouped together hierarchically in NVivo and printed out where annotations were made by hand to help generate themes. The analytical theme generation process aimed to extend beyond the original study themes and was reviewed by the second author.

### Quality assessment

The methodological quality of the included studies was assessed using the appraisal tool CASP Qualitative Studies Checklist (Critical Appraisal Skills Programme, 2018) (Table 1). The ten appraisal questions focus on research design, recruitment method, data collection, researcher/participant relationship, ethical considerations, data analysis, clarity of findings, and importance/value of the research. The authors of the checklist did not recommend scoring up the results but instead emphasised using the appraisal tool qualitatively. SHY completed the CASP and this was checked by US. The quality of the studies did not particularly impact on the theme generation, but instead provides context for the overall analysis.

**Table 1:** CASP quality appraisal table

		Aim of research	Methodology	Appropriate research design	Appropriate recruitment strategy	Appropriate data collection	Reflexivity	Ethical issues	Rigour of data analysis	Clear research findings	Research value
1	Chang et al. 2020	yes	yes	yes	yes	yes	yes	yes	yes	yes	yes
2	Chan and Ma, 2005	yes	yes	yes	yes	yes	Lacks researchers’ reflexivity. Slightly uncertain whether that would impact the power and relationship when the researcher had lunch with the participant.	yes	yes	yes	yes
3	Chan & Ma, 2008	Yes – although slightly unclear	yes	yes	yes	Unclear data collection method	Researcher who analysed the transcripts was the therapist which may bias interpretations of findings.	Not mentioned	Unclear analysis method	yes	yes
4	Lee et al. 2021	yes	yes	yes	yes	yes	yes	yes	yes	yes	yes
5	Ma & Lai, 2006	The aim of the study could be written in a clearer way	yes	yes	yes	yes	The analysing researcher was also the therapist for some of the participants, Lacks researchers’ reflexivity	Consent was mentioned. Ethics approval not mentioned	Yes	Yes	yes
6	Ma, 2008	yes	yes	yes	yes	yes	Researcher who analysed the transcripts was the therapist which may bias interpretations of findings, Lacks researchers’ reflexivity	Consent was mentioned. Ethics approval not mentioned	yes	yes	yes
7	Ma, 2012	yes	yes	yes	yes	Data were not all transcribed verbatim	Researcher was the therapist, which may bias interpretations of findings, Lacks researchers’ reflexivity	yes	Unclear explanation of the analysis method	yes	yes
8	Ma et al. 2012	yes	yes	yes	Only included 3 northern Chinese provinces	yes	No – lacks researchers’ reflexivity	Yes	Only summarised the conceptualisations of the interventions without any analysis	yes	yes
9	Ma et al. 2021	yes	yes	yes	yes	yes	Lacks researchers’ reflexivity	yes	The analysis method and results did not match (Braun and Clarke cited but mentioned percentages)	yes	yes
10	Sun et al. 2019	yes	yes	yes	yes	yes	yes	yes	yes	yes	yes
11	Wu and Chen, 2021	yes	yes	yes	yes	yes	Unsure what the sentence ‘researcher himself was a tool for collecting data’ means	yes	yes	yes	yes
12	Wu and Harrison 2019	yes	yes	yes	yes	yes	yes	yes	yes	yes	yes

### Reflexivity

It is important to be aware of researchers’ biases and positionality in qualitative analysis. SHY is Chinese by ethnicity and was born and raised in Hong Kong. She completed her undergraduate and postgraduate studies in the UK and works in the National Health Service in the UK as a clinical psychologist, where intrinsically western and white-orientated models were taught and practiced. Therefore, she is aware of her background where on the one hand, she understands culture-specific issues in some parts on EA, on the other hand, she is in a slightly detached position professionally and geographically. US is a UK-trained psychiatrist who is originally from Germany and has extensive experience in EDs. She approached the research topic and data from the point of view of an EDs expert as well as using her experience of treating EDs patients from East Asia in the UK as well as collaborating with East Asian researchers. She is aware of her positionality as a White European woman and this allows her to discuss the cultural differences between East and West with SHY.

## Results

**Fig. 1:**
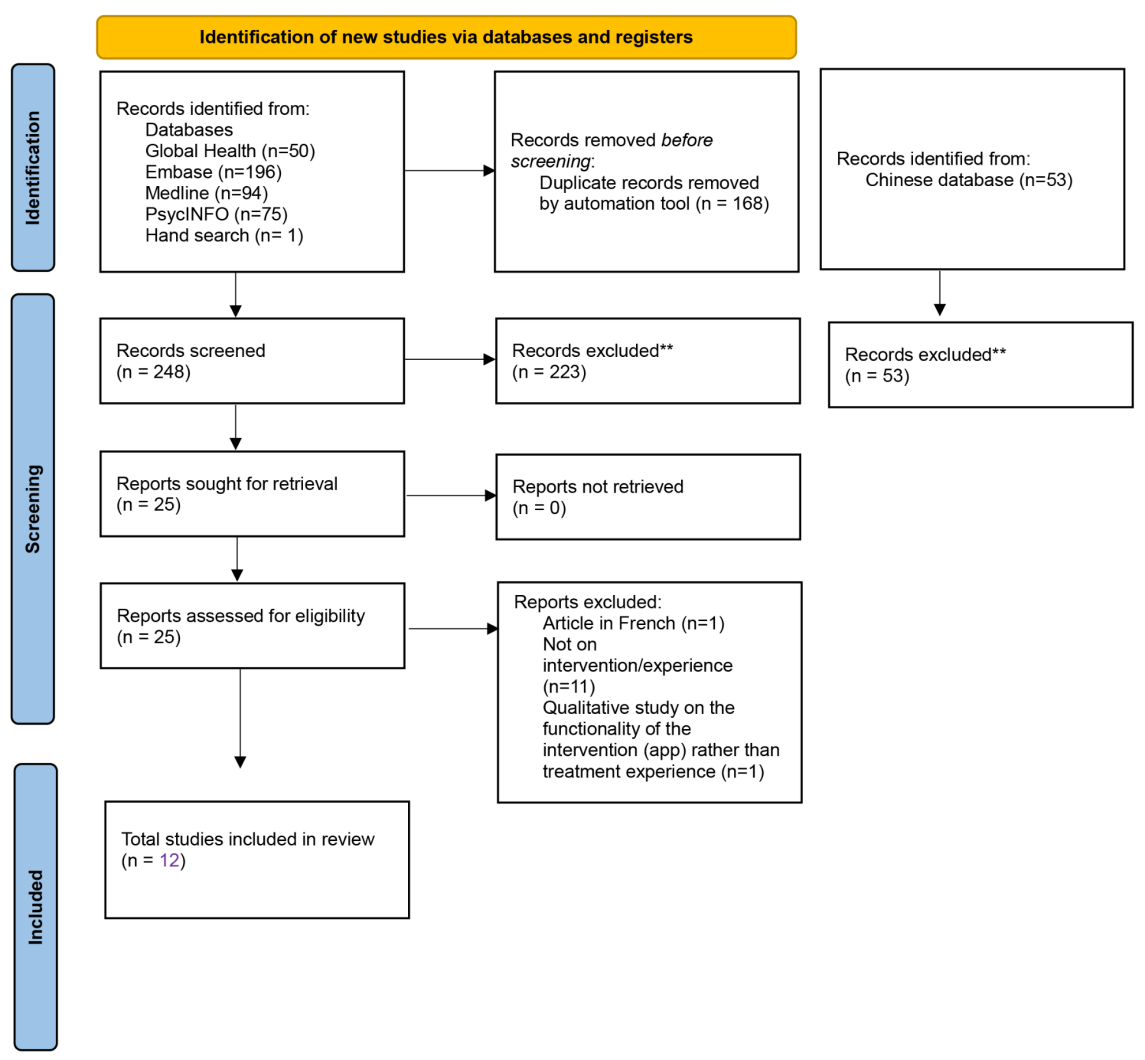
PRISMA flowchart

A total of 12 studies were included. However, two of the studies (Ma and Lai, 2006; Ma, 2008) were based on the same cohort of participants. In one of these papers, the research focus was on perceived treatment effectiveness, and in the other on experiences of treatment. Figure 1 shows the PRISMA chart. None of the Chinese language studies were qualitative studies on experiences of EDs interventions and hence all included studies were in English. Table 2.1 & 2.2 shows a summary of the study characteristics and extracted settings and themes. Overall, most studies examined people with AN except for [26] who included people with BN, purging disorder and night eating disorder, and [27] who included BN. One study examined parents’ views and perceptions of help for AN in Hong Kong [28], and two studies examined professionals’ views and perceptions of treating young people with AN in Taiwan [29]; [30]. The mean age of the participants interviewed was below age 30 for all patient-related studies. All studies were conducted in Chinese-speaking (Cantonese and Mandarin) regions of EA. The majority of the patients interviewed identified as females – one out of 69 participants in total across all studies identified as male.

**Table 2.1:** A summary of the study characteristics and extracted settings and themes (views from patients/ parents)

	Paper	Location	Age and gender	*N*	Diagnosis	Setting	Type of therapy	Theme	Method
1	Chan and Ma, 2005	Hong Kong	Mean Age = 21 Female	*N* = 1	AN	Not mentioned	Family therapy	Five themes emerged from the patient’s storied review of the sessions: (1) how she viewed her own personal characteristics; (2) her changed perceptions of her family relationships; (3) her perceptions of her family quarrels’ dynamics; (4) her resistance to recovery; and (5) her significant events in family therapy.	Case study Phenomenological study using the Feminist epistemology. Interpretative paradigm, where the patient served as a research instrument and was invited to review six family sessions and came up with themes herself
2	Chan & Ma, 2008	Hong Kong	Mean Age = 21 Female	*N* = 1	AN	OP	Family therapy	Six themes were generated: (1) “Only my mother is concerned about me” (2) “I am not Alone in this Battle (3) “My Family Will Always Stand by My Side” (4) “I am Really Jealous of My Sister” (5) “Just Because We Are Siblings” (6) “The Therapist Really Cares about Me”	Case study Analysis method was not specified (‘observation’ and ‘analysis of the transcript’ was mentioned)
3	Lee et al. 2021	Taiwan	One was born in 1980s (exact age unknown), one was 28, one was 27 Females	3	AN	Not specified	Faith-based counselling and unspecified therapy type	1) anorexia as a function of the conflictual bicultural self; 2) recovery as a pathway towards an integrated bicultural self; and 3) the paradoxical roles of Chinese cultural heritage in anorexia and recovery.	Multiple-case qualitative method; semi-structured interviews conducted by the first author in Mandarin Chinese The interview questions were designed to explore the participants’ recovery processes and to further gauge the interaction between participants’ own cultural characteristics, backgrounds, and values and their individual recovery experiences. Thematic analysis
4	Ma & Lai, 2006	Hong Kong	Mean age at referral was 14 Mean age for the adults was 23.4 Females	Adolescents (*n* = 18), adults (*n* = 6) with AN and their parents (n of parents not reported)	AN	OP and IP	Family therapy	Perceived Helpfulness of Family Treatment on Symptoms; Enhancing Patients’ Psychological Well-Being Through the Therapist’s Care and Family Support, Lowering the Parents’ Psychological Distress, Empowering Parental Coping Through Empathetic Understanding, Support and Broadening Conceptualisation of Problems, Reduced Family Conflicts and Increased Family Cohesion.	Semi-structured interview Analysis method was not specified, ‘thematic summaries’ was mentioned
5	Ma, 2012	China	Age was not specified, 4 were students (primary school to college), and one was a working adult. Gender 4 females, 1 male	Five families	AN, BN	OP	Family therapy, duration of treatment ranged from 6 months to 2 years	Two emerging themes in relation to the perceived changes were identified: (1) the perceived contextual changes, including the involvement of the previously disengaged father in the care, and the change in the parenting methods, and (2) the different roles played by the young person, the parents, and the therapist in healing	Case study as the method to identify the symptomatic cycle of family interactions that have maintained the disorder, to track the process of the therapeutic change, and to understand the roles of the family and the therapist in effecting such changes Post-treatment semi-structured interview (an hour). Content analysis
6	Ma, 2008	Hong Kong	Mean age at referral (adolescent) = 14 Mean age (young adult) = 23.4 Female	*N* = 24 (18 adolescent, 6 young adults)	AN	IP/OP	Family therapy	The narratives that have emerged have facilitated hearing the clients’ and their family members’ voices, especially in the areas of (a) perceived concepts of family therapy, (b) the perceived therapeutic relationship and its linkage to positive change, (c) perceived intervention strategies as employed in family treatment, and (d) the participants’ own role in problem-solving.	Post-treatment interviews on perceived helpfulness of treatment and the perceived functions of the therapist. Content analysis
7	Ma et al. 2021	China	Median age from people who sought treatment = 23 Females	*N* = 31	AN, BN, OSFED (Did not specify the number of those who took part in the interview)	IP, OP	Mixed	Themes emerged from interviews revealed positive inpatient treatment experiences for anorexia nervosa, but negative outpatient treatment experiences, unaffordable care, and ineffective psychopharmacological treatments. Parents, friends, and partners were sources of social support, but participants largely felt misunderstood or blamed by these same entities. Shame, not recognizing ED as an illness, and financial constraints were listed as the primary reasons for not seeking treatment.	Recruit via WeChat Interviewees chose to provide their qualitative data via semi-structured telephone interviews (*n* = 19; 61.3%) or in writing (*n* = 12; 38.7%) on treatment/ help-seeking experiences, as well as reasons for not seeking treatments. Thematic analysis with codebook template
8	Sun et al. 2019	Hong Kong	Parents Age 49–57 4 females 2 males Patients were all female	6 families	AN	Mixed	Mixed	Barriers 1. Perceived limited empathy from doctors. 2. Fear of admission to psychiatric hospitals 3. Health professionals’ limited knowledge on AN 4. Limited explanation of the treatment approach. 5. Insufficient communication between parents and health professionals. 6. Inadequate understanding of parents’ role in managing AN 7. Insufficient coordination among health professionals 8. Insufficient treatment options Enablers 1. Inpatient care in paediatric wards. 2. Assistance from patient support organizations. 3. Supportive school environment 4. Empathetic health professionals. Reflections from parents 1. Management approach of parents The need for psychological support	Focus groups (using semi-structured interviews) were conducted on parents of adolescents with AN recruited through an eating disorder association in Hong Kong to understand their views and experiences regarding the help-seeking and treatment process Content analysis
9	Wu and Harrison 2019	China	Aged 16–19 Females	4	AN	IP	Inpatient treatment (mixed)	Four master themes emerged from the data: 1. participants’ understanding of the nature of the treatment received, - Subordinate Themes 1: Discrepancies between the dominant focus of physical improvement at the expense of the psychological support desired by participants - 2: Inadequate professional psychological support - 3: Excessive use of medication and its effects - 4: Use of tablet computers as a component of treatment 2. peer influences during the admission, - Subordinate Theme 1: Positive Peer Influences - 2: Negative Peer Influences 3. the impact of treatment on wellbeing and - Subordinate Theme 1: Physiological and behavioural improvements - 2: Limited psychological or cognitive benefits - 3: Short-term impacts of treatment - 4: Psychological impact of intervention methods on wellbeing 4. participants’ sense of self. - Subordinate theme 1: Self-perception - 2: Confidence in ‘normal’ living and eating - 3: Confidence in controlling disordered eating in the future	Semi-structured interview (30 min) IPA

**Table 2.2:** A summary of the study characteristics and extracted settings and themes (views from health professionals)

	Paper	Location	Age and gender	*N*	Diagnosis	Setting	Type of therapy	Theme	Method
1	Chang et al. 2020	Taiwan	Mean age = 30.2 Physicians: all males Dieticians and nurses: all females	*N* = 16 healthcare professionals, including 10 nurses, 3 dieticians and 3 physicians from the paediatric ward	AN	IP	Paediatric medical stabilisation	Five themes and ten subthemes were identified: 1. Building a trusting relationship first: (a) spending time to build trust with the client and (b) establishing a relationship with the client’s parents; 2. The key to treatment success: (a) Clients’ awareness of the illness and (b) parents’ support for clients; 3. Consistency of team treatment goals: (a) maintaining stable vital signs and (b) achieving caloric intake; 4. Empowerment with knowledge about anorexia: (a) continuing education for healthcare professionals and (b) interdisciplinary collaborative care; and 5. Using different interaction strategies: (a) the hard approach and (b) the soft approach.	Semi-structured interview (45–60 min) on service providers’ experiences in the treatment and care of adolescents with AN Qualitative content analysis approach
2	Ma et al. 2019	China	Mean age = 44.56 Female (*n* = 32); Male (*n* = 9)	*N* = 41	Bulimic-spectrum disorders	Varied	Varied	Two participants did not mention bulimic symptoms in their conceptualisations Interventions described include CBT, art therapy, cognitive intervention, medication, relaxation and mindfulness, rational emotive therapy, art of midwifery, narrative therapy, and Yi Xiang Dui Hua (image talk).	Semi-structured interviews of a hypothetical case involving vomiting and binge eating to get their views on case conceptualisation using purposeful sampling of therapists from three Northern Chinese Provinces Directed content analysis
3	Wu and Chen, 2021	Taiwan	Mean age = 30 Females	Nursing staff from the paediatric ward at a university-affiliated medical centre, *N* = 10	AN	IP Paediatrics	Paediatrics. Length of hospital stay is about 3–4 weeks	Three themes with eight subthemes: (i) Struggling to develop therapeutic relationships. - High defensiveness and unwillingness to disclose - Rigid and indifferent interactions Emotion triggers: diet and weight - Harsh handling affecting the relationship between nurses and patients (ii) Selective focusing, - Insufficient time - Inability to provide quality nursing care (iii) Difficulty changing minds - Passive treatment Cognitive distortion of body image	Semi-structured interviews on experiences of nursing staff with adolescents with AN. Conventional content analysis

### Themes

Three main themes were identified.

#### Theme 1. Delineating physical and psychological recovery

People with lived experience of EDs described how treatment ‘*was helpful but [they were] not symptom free*’ ( [26]. In particular, participants often described the distinction between physical health and their psychological health, suggesting that recovery involves both components and that (inpatient) treatments seem to only support physical recovery. A participant noted, ‘*the only positive impact was physical health*,* others (were) all negative; but without that I would have already died.’* [31]. Another participant concurred, *‘I did not find the staff helped me with my anxieties about my weight…I was not helped psychologically*,* it was all about the physical improvements’* [28]. However, without psychological recovery, participants described their symptoms worsened post-discharge. For example, participant said *‘I think it [bingeing and purging becoming even worse after discharge] might be because I have gained lots of weight during the period of receiving inpatient care*,* but I could not psychologically accept it…thus…I started to fast badly*,* and after a while*,* my bingeing emerged and my urge to eat got even stronger.’* [28]. This view was shared among professionals as well. One dietician in Taiwan reflected that “*We should study psychology. Anorexia is not only physical’* [29].

On the other hand, there are other participants who described a full recovery (‘*[I] live like a normal person’* [28].

#### Theme 2. ‘I am not alone in this battle’

This theme includes three pairs of relational dyads – the patients in relation to their families, their therapists, and their peers.

#### Sub-theme 2.1. The dialectics of interdependence and independence

In the included studies, it appears that certain cultural values of interdependence and filial piety may provide a fertile ground for EDs to develop. As a participant (person with an ED) put it,

*‘I wanted to have some freedom from my parents but I didn’t want to go against them. Their control/protection was benign*,* good for me*,* but it’s seamless and suffocating. I just need some space to make my own choice. Anorexia was part of my identity because eating and weight are the only things I have control over’* [4].

Mealtimes are seen as a non-negotiable duty especially if the older family members prepare the dishes. As mentioned by a Taiwanese woman with lived experience of an ED: ‘*mealtime was held to be sacred*,* reflecting the Chinese belief that eating works towards preserving harmony*,* cohesion*,* and unity in the family. Grandmother’s cooking and food serving signified her devotion to*,* and affection for*,* her children. The entire family was*,* in turn*,* expected to reciprocate their grandmother’s gesture by observing filial piety and obeying her rules about food and meals’* [4].

For some participants, the need to obey senior family members, fulfil family duties, and prioritise others’ needs may suppress their own needs and lead to internal conflicts: ‘*I should get more involved with my parent’s business*,* care more about how they feel and what they want’* [4]. The researchers speculated that this may also relate to traditional Chinese culture, where males are more valued than females, and daughters feel the need to live up to the family’s expectations when they are an only child. In these situations, healing involves individuation from interdependence and exploring self-identity to prevent relapse [32]. This quote illustrates this point: ‘*as I started seeing myself independent from my mum*,* I became more comfortable and no longer felt inferior to her… my bingeing and purging frequency reduced.’* [4]. Another participant from the same study described moving out of the family home as a turning point towards EDs recovery [4].

Nevertheless, the cultural value of interdependence can also serve as a protective and motivating factor towards recovery. Instead of citing personal reasons for recovery, some participants described their motivation to get better for their parents, influenced by the cultural value of ‘saving face’: ‘***…****My anorexia was a face-losing thing…I felt like becoming too much a burden…I was eager to become normal again…so that I could save face for my parents’* [4]. When a participant looked back on the recovery, one discussed the cultural value that emphasises ‘the body is given by the parents’: ‘*I vomited the money you earned…. I hurt the body you have given me…again and again’* [26].

Similarly, recognising that the family will unconditionally accept them regardless of whether they manage to meet their parents’ expectations, can also be motivating. One participant described how her family will always stand by her side,

*‘I really decided to walk out of this eating disorder swamp. I felt that*,* no matter what*,* my parents would love me*,* even when I’m vomiting and when I am the ugliest. Perhaps they couldn’t understand me*,* but because that’s me*,* they would accept this person unconditionally’* [26].

Therapy provides a space for the family to ‘*have a deeper chat’* and to facilitate a greater understanding of each other, improving the family relationships. This includes both the parent-child dyad as well as sibling dyad:

*‘The therapist did not talk much about eating in treatment. She worked on the family relationships. Let’s understand her work in this way. With the onset of the illness*,* the family must have problems and the family relationship must be damaged… when our communication improved and our relationships were repaired*,* we became more harmonious and the child would listen to other parents.’* [27].

*‘In fact*,* I can see that both my brother and sister want to help me*,* but I can’t accept the way they help me…now I can see that they just want to give some ideal solutions to me.*’ [33].

Some studies emphasise the role of the father and increasing paternal presence (e.g., [27]). Traditionally, it is assumed that mums are responsible for domestic matters as well as the children’s wellbeing. As a mum put it, *‘[the child’s] father is a CEO of a huge company and I don’t want to upset him. I want him to concentrate his energy and time on work. I told him about my difficulty only when I could no longer handle it’* [27]. A father reflected on his guilt towards not caring for his daughter: *‘… I should stay behind to take more care of her’* [27]. Therapy plays a pivotal role in fostering and enhancing the father’s presence, while also illuminating the daughter’s longing for paternal care. In a case study, Lily, a participant, reported that her improved relationship with her father facilitated a return to normal and regular eating habits. As a result of therapy, her father began dedicating more time to the family, acknowledging that he previously prioritised rest over spending time with family. In another scenario where the individual’s father had passed away, the therapist emerged as a dependable father figure, providing invaluable support and understanding [4].

#### Subtheme 2.2. Clinician as a trusted and safe base

Both clinicians and patients described important common factors in therapy such as calm, patience and building trust. In the paediatric wards, the nurses mentioned “*You must take the time to establish a relationship with her. She is willing to rely on you*,* and she is willing to tell you where the problem is*.” [29]. A patient mentioned *‘the therapist has really good temper. No one can stand to talk to me so long*,* except my mother*,* including my brother and sister. And her tone makes me think that she’s a person I can trust.’* [34]. Developing a safe base allows the families to then explore more difficult topics. Studies describe the use of the word ‘*as a bridge’* to recount the role of therapist in treatment:

*‘She made me feel confident. We began to trust her (the therapist). We felt that she can help us. With that trust in mind*,* I feel free to disclose my feelings honestly…my body weight dropped and I was very frightened….I had no confidence and was very fearful. She (the therapist) looked at me with a warm smile and in a firm tone*,* said that she had confidence in me and I could make it’* [34].

#### Subtheme 2.3. Relating to peers with EDs

References to sharing and comparing EDs behaviours, such as sharing purging techniques, were noted [28]. For instance, one participant described observing peers using their iPad to calculate meal calories and researching diets online to lose weight after discharge. In the analysis, the authors hypothesised that due to the historical One-Child Policy in China, being on the ward might be the participants’ first time living with peers away from their families. They wondered whether some of the group dynamics of cooperation and conflict might be attributed to the lack of experience of living with siblings.

On the other hand, positive aspects from peers were also noted, such as finding people to talk to: ‘I had been keeping this secret (my ED) for an extremely long time without finding somebody to talk to’ as well as reducing vomiting behaviours due to others reporting to the nurses [31].

#### Theme 3. Barriers to change

Four aspects of barriers were described: financial, structural, coercive practice and cultural.

#### Subtheme 3.1. ‘I am wasting my family’s money’

One participant mentioned, “Psychotherapy or counselling would cost me 400/500 yen (approximately 70 USD) per session. I am still a student and don’t have much money. I thought I could follow self-help resources and treat myself” [26]. While she expressed an individual perspective, others described, “We are not wealthy as a family,” indicating a family-oriented viewpoint among the participants. For instance, one participant discussed how their family did not consider finances a barrier to treatment:

*‘…I can see that my family doesn’t care about money when compared to my health*,* and my sister also wastes her study time to keep on seeing the therapist every week. Now I can see that they all treat me very*,* very well*,* and want me to be healthy again.’* [32].

#### Subtheme 3.2. Unavailable professionals and services

Participants were dissatisfied by the lack of specialist services, as well as the lack of knowledge of EDs among healthcare professionals. This is evident in terms of the short period of time they are being seen for:

*‘The diagnostic process involved me describing my situation and the doctor asking me more questions…diagnosed me with bulimia nervosa. The whole process took about 6 to 7 minutes. It was very short and nonspecific. I feel my condition was not taken seriously.’* [26].

The scarcity of specialist services was mentioned by multiple participants. One of them said, ‘*treatment resources are only available at big hospitals’* in mainland China [26, 35]. In Hong Kong, parents described how difficult it was to find therapists that are knowledgeable about AN:

*‘I really don’t know where you could find family therapists that specialise in treating anorexia in Hong Kong… in foreign countries*,* there is usually a team which put strong emphasis on family support and teamwork*,* and such kind of support is totally unavailable in Hong Kong’* [28].

The lack of knowledge among professionals can also lead to patients and families feeling invalidated. A doctor mentioned that amenorrhoea could be stress-related and could be a common gynaecological issue, or patients were told to use willpower to overcome their EDs. Parents expressed feeling blamed:

*‘During the consultation*,* we were scolded by the psychiatrist [in A & E]. Have I done anything wrong? He told me that my daughter was well-behaved but I left her in other people’s care. Hey*,* I have to work! I have already tried my best to find something that is suitable for my daughter.’* [28].

These experiences by parents are echoed by professionals in Taiwan, who acknowledged their treatment knowledge gap:

One physician said, “*Our care for anorexia is taught by the attending physician one by one*,* from the intensive care unit to the ward care*,* and then to the outpatient care. In fact*,* education is carried out during the follow-up process and the ward rounds. This kind of education only means that the few people who are cared for know how to take care of them. Nurses still don’t know how to care of them”* [29].

The other gap acknowledged was the lack of awareness of non-AN EDs. In a study where a hypothetical vignette of a female who vomits and binges were presented, researchers noted that almost every clinician in the study specified AN rather than BN [35].

#### Subtheme 3.3. Coercive practice

Coercive practices, particularly within inpatient settings, were reported, involving the use or threat of restraints and nasogastric (NG) tubes. For instance, a nurse mentioned that even the visible presence of an NG tube could be employed as a form of coercion [29]. Describing their own experience as a former inpatient, one individual expressed deep distress regarding witnessing physical restraints [28]. Such experiences resulted in negative treatment experiences, with participants recounting psychological trauma and nightmares related to their inpatient care [28]. In outpatient family therapy, mothers described feeling like a ‘villain’ and needing to force feed their child [28]. In view of such practices, participants expressed that such treatment compelled them to act against their desires, and they doubted its efficacy in addressing their weight-related fears [32].

#### Subtheme 3.4. Converging and diverging cultural ideals

While thin ideals are often valued in EA cultural norms, there are also contrasting views that perceive thinness as a Western ideal. Participants in the study perceived being chubby as the ideal in Chinese culture, as one individual expressed: “In our culture, being chubby should mean pretty and lucky. My first memory of the really thin women were western models and movie stars…my mum always said they are ugly” [4]. This contradicts the thinness ideal highlighted in other studies (e.g. 11). Interestingly, exposure to the actual environment in the West helped correct participants’ perceptions of body ideals, which proved beneficial to their recovery:

*‘[the participant] highlighted that these cross-cultural exposures and experiences living abroad had enabled and empowered her to challenge the stereotyped images of beauty portrayed and perpetuated by western media… “after I moved to the US*,* I realised that people here do not look like those in the movies…” ’* [4].

### Study quality

Most studies used adequate qualitative methodologies. The main quality issues identified include not mentioning ethical considerations, lacking researcher reflexivity, lacking details regarding the analytic steps, and that in some studies (e.g. where family therapy was the treatment modality), the analysing researcher was also the treating therapist, which may introduce bias (see Table 1 for more detail).

## Discussion

The 12 studies included in the review generated three analytical themes in response to our research question on people’s experiences of treatment in East Asia (Table 3). Cultural aspects relating to people’s experiences were considered when identifying themes.

**Table 3:** Summary of themes

Themes	Subthemes
Theme 1. Delineating Physical and Psychological Recovery	/
Theme 2. ‘I am not alone in this battle’	Sub-theme 2.1. The Dialectics of Interdependence and Independence Subtheme 2.2. Clinician as a trusted and safe base Subtheme 2.3. Relating with peers with ED
Theme 3. Barriers to change	Subtheme 3.1. ‘I am wasting my family’s money’ Subtheme 3.2. Unavailable professionals and services Subtheme 3.3. Coercive practice Subtheme 3.4. Converging and diverging cultural ideals’

A diverse range of treatment was described - including family therapy, paediatric/ psychiatric inpatient care, cognitive behavioural therapy, and faith-based counselling. This contrasts with the systematic quantitative scoping review by Yim & Schmidt [16], where CBT and internet interventions were the main treatments in focus. Some of the themes share similarities to other qualitative syntheses on AN treatment such as improved family relationships as well as the perceived authoritarianism and control in treatments [18], and the use of restraints and NG tube in inpatient wards. Similar to the findings from Yim & Schmidt [16], participants also directly mentioned financial barriers and the unavailability of specialist professionals/ services.

The current review goes beyond the cultural adaptations described in Yim and Schmidt [16]. More nuanced factors such as family roles, cultural values and norms were shared by participants, which can be important issues to be addressed in therapy. With respect to policy, the historical One Child Policy (OCP) in mainland China was mentioned in Wu and Harrison [28] where they hypothesised that this could potentially impact the interpersonal dynamics in inpatient settings. This was not mentioned in other studies in Yim & Schmidt’s [16] review. Whether or not the OCP affects the social literacy of single children is under debate, as the single child will still be interacting with peers at school [15]. This is also potentially confounded by the nature of EDs where body comparison is part of the symptomatic behaviour. It is difficult to disentangle the relative influences on people’s negative experiences in inpatient treatments. In contrast, the impact of OCP is wide-ranging and other impacts may influence the development or maintenance of an eating disorder. OCP has led to an imbalanced sex ratio with more males to females in China and having one child only may be seen as a deprivation of one’s reproductive choice. This also adds to the pressure of looking after one’s elderly parents without the support of other siblings. At the same time, single children (especially girls) faced immense pressure to excel, and are enrolled in multiple tutorials and extracurricular activities [16]. The pressure to achieve, in addition to preserving the family’s ‘face’, may contribute to the development of an ED [26]. Relating to the negative aspects of peer influence in EDs wards, it would be useful to explore if similar issues were found in group therapies. Future studies could also explore how single children versus non-single children perceive group or residential treatments (i.e. where there are the same rules for all).

Collectivist culture, where family harmony and ‘saving face’ are esteemed [4], can present a complex dynamic. Whilst this cultural value may impede help-seeking due to stigma, participants also noted that it functions as a motivator for getting better. Another significant cultural value is Filial Piety, where researchers speculate it may hinder patient’s individuation process [7]. The necessity for individuation becomes evident as participants highlighted pivotal moments in their ED recovery, such as moving out of the family home or moving abroad for studies [4]. Initially, participants with EDs struggled with parental expectations and prioritised family wishes over personal aspirations. For some, their EDs may serve the function of creating distance/ challenging parental control or wishes without overtly going against them [4]. This is potentially compounded by cultural beliefs favouring men over women, leading girls to internalise feelings of inferiority. Balancing familial and individual needs emerges as a central focus in EDs therapy for them. However, similar to other culture-specific values, filial piety can potentially also be a protective factor, motivating patients to comply with parental directives and attend therapy. The idea of interdependent self-construal is pertinent here [14]. Patients described relational motives to recovery, such as ‘I am “vomiting” your money and your love’. The process of individuation also includes maintaining family connections. Echoing findings by Medway and Rhodes [18], some family therapy studies in East Asia (e.g., [27] underscore the reorganisation of family dynamics and roles, often with increased paternal involvement. Yim & Schmidt [17] speculated that CBT was preferred to family therapy due to most parents working full-time in East Asia. This sentiment is reflected in some parents’ statements like ‘Hey, I have to work!’ However, the present review suggests that the benefits of family therapy are being recognised for restructuring family dynamics and roles, as well as increasing communications and bonding. This is evidenced in the theme ‘I am not alone in this battle’, where family relationships are perceived as improved, and families come together and the patient did not feel judged or uncared for by their parents. This agrees with Tan et al’s [20] view of using a maternalistic approach in treating ED patients in Asia.

### Clinical recommendations

This review, along with Yim and Schmidt (2023), identified treatment, training and research gaps for EDs in EA. We propose the following clinical implications and recommendations:

#### EDs conceptualisation in EA

Clinicians in EA need to have greater awareness of EDs in general, especially EDs other than AN [35]. Although our combined reviews show that individual treatment approaches seem to be the norm in EA, it will be useful to include the family context as part of the formulation and treatment planning.

Clinicians should have an awareness of how culture relates to one’s formulation of an ED whilst attending to individual differences. Some examples of culturally informed treatment planning may include harnessing the interdependence and cultural norms of ‘sacred’ family meals as an act of care rather than the family being cast in the role of a ‘villain’. It may be appropriate to consider both interdependent, relational motivators and goals, in addition to personal goals towards recovery, paying attention to the process of individuation whilst maintaining connectedness.

#### The role of body image

Body image ideals appear to be another conflicting value. On the one hand, studies mentioned how thin ideals are pervasive in EA (e.g. 13), which could be an influence from Westernisation. On the other hand, participants described being ‘chubby’ as being valued [4]. Whilst there may be generational differences in body ideals, it could also create a sense of internal conflicts if young people’s perceived ideals are different from those of their parents. With the conflicting findings from the studies regarding the relative influence of Western and Asian media (e.g. [12]), it is important for clinicians to consider a multidimensional conceptualisation of body image and not to make assumptions around the body ideals that the individual is influenced by. Moreover, it may be important to include the family’s perception and ideals of the person’s weight and shape.

#### Capacity building

The advancement of telemedicine can facilitate better more in-depth training of medical professionals on understanding and treating EDs (e.g. see [36], as well as increasing the affordability and accessibility of treatments, and also capacity building of evidence-based EDs treatments in EA. It is recommended that journal special issues, conference themes on culture and EDs, or special interest groups/ clinical research networks on EDs in East Asia/for East Asians should be organised to facilitate knowledge and skills exchange.

### Limitations

All the included studies are conducted in the Chinese (Mandarin and Cantonese)-speaking regions in EA. Our search strategy did not include grey literature which is a limitation. Some researchers may argue that qualitative studies are context specific and a synthesis of such findings may de-contextualise them. Whilst the aim of this review is not to provide generalisability, it is worth acknowledging that in terms of context transferability, people’s experiences and views in other regions such as Japan and Korea are unknown. It may be that relevant papers were written in the respective languages and therefore not found in our search. Nevertheless, the settings and populations of the included studies were listed in Tables 2.1 and 2.2, which could assist in the interpretation of the transferability of the findings.

## Research recommendations

Most of the EDs study participants experienced AN in the studies, and little is known about the experiences of people with BN, BED, or the relatively newer ARFID diagnosis in the region. This is especially pertinent as the prevalence of BED and BN is higher than that of AN in China [2].

The prevailing models of treating AN in the West such as ED-focused family approaches for adolescents, are also an underexplored area, so we could not identify whether there are differences in people’s experiences or perceived effectiveness of an ED-focused therapy versus the modified Micucci’s model. The concept of non-fat phobic AN was not mentioned in the studies. Moreover, the studied populations were relatively young (most of them were under 30). Future research on older individuals with EDs in EA would be valuable.

Gender is another key area that needs to be addressed. Across all the included studies, only one patient identified as male. Given most of the studies identified were conducted in China, and that China has a larger male to female ratio, the finding is therefore somewhat surprising. It is difficult to understand how gender and its intersection with aspects of EA culture may influence treatment experiences.

In terms of methodology, it is important for future research to consider researchers bias and reflexivity to increase transparency, credibility and research rigor.

Given that professionals may perceive EDs as a gastrointestinal or gynaecological issue, it is likely that EDs are under-detected within those specialities. Future explorations of specific cultural factors and the relative influence of different body ideals are needed, and understanding the unique cultural struggles of the East Asian Diaspora versus East Asians residing in their home countries.

### Electronic supplementary material

Below is the link to the electronic supplementary material.

Supplementary Material 1

## Data Availability

No datasets were generated or analysed during the current study.

## Abbreviations

EA: East Asia
ED(s): Eating disorder(s)
AN: Anorexia Nervosa
BN: Bulimia Nervosa
BED: Binge Eating Disorder
