# Supplementary material for: Views and experiences of eating disorders treatments in East Asia: a meta-synthesis
Source: J Eat Disord. 2024 Aug 20;12:120. doi: 10.1186/s40337-024-01070-4 (PMC11334303; doi:10.1186/s40337-024-01070-4)
Supplement: Supplementary file 1 — Supplementary Material 1 [file 40337_2024_1070_MOESM1_ESM.docx]

**Supplementary information**

**Example of search on one database**

| 1 | (eating disorder* or bulimia or anorexia or binge eating or disordered eating or ARFID or Avoidant Restrictive Food Intake Disorder).ab,hw,kf,ti. |
| --- | --- |
| 2 | (China or Hong Kong or Taiwan or Macau or Macao or Mongolia or Japan or Korea or Chinese or Taiwanese or Mongolian or Japanese or Korean or east Asia or east Asian or far east).ab,hw,kf,ti. |
| 3 | (qualitative or interview*).ab,hw,kf,ti. |
| 4 | eating disorder.mp. or exp "Feeding and Eating Disorders"/ |
| 5 | exp Asia, Eastern/ |
| 6 | exp Qualitative Research/ |
| 7 | 1 or 4 |
| 8 | 2 or 5 |
| 9 | 3 or 6 |
| 10 | 7 and 8 and 9 |
| 11 | limit 10 to (chinese or english) |
| 12 | limit 10 to (autobiography or bibliography or biography or clinical conference or clinical trial protocol or comment or congress or consensus development conference or consensus development conference, nih or dataset or dictionary or directory or duplicate publication or editorial or electronic supplementary materials or letter or "review" or "scientific integrity review" or "systematic review") |
| 13 | 11 not 12 |
